# Long-Term Environmental Durability of Recycled Textile-Low-Density Polyethylene Composites Reinforced with Plant Fibers: Mass Uptake, Flexural Stability, and Microbial Colonization

**DOI:** 10.3390/ma19214504

**Published:** 2026-10-26

**Authors:** Marcin Małek, Norbert Lipka, Wiesław Kaca

**Affiliations:** 1 Faculty of Civil Engineering and Geodesy, Military University of Technology, Generała Sylwestra Kaliskiego 2, 00-908 Warsaw, Poland; 2 Department of Microbiology, Institute of Biology, Faculty of Natural Sciences, Jan Kochanowski University in Kielce, Uniwersytecka 7, 25-406 Kielce, Poland; norbertlipka@wp.pl (N.L.); wieslaw.kaca@ujk.edu.pl (W.K.); 3 VIVE Textile Recycling Sp. z o.o., Łopuszańska 22, 02-220 Warsaw, Poland

**Keywords:** recycled textile composite, low-density polyethylene, plant fibers, durability, flexural properties, water uptake, biodeterioration, microbial colonization, plastisphere

## Abstract

**Highlights:**

- One year of exposure to soil or water resulted in a mass gain of only 0.12–0.58%.
- Flexural properties changed by no more than 1.1% after 12 months.
- Viable bacterial and fungal communities persisted on the composite surfaces.
- Mechanical stability coexisted with high abundances of genera associated with cellulolytic and ester-hydrolytic functions.

**Abstract:**

Recycled textile-polyethylene composites combine heterogeneous polymer and cellulosic phases whose long-term response to moisture and microbial colonization determines their suitability for building products. To the best of the authors’ knowledge, this is the first study to couple one-year, real-environment (non-accelerated) exposure of product-scale composites made from mixed post-consumer textile waste with mechanical testing and amplicon-based profiling of the viable surface microbiota, thereby linking material durability and biological colonization in a single dataset. In this study, recycled textile-low-density polyethylene (LDPE) composites produced by intrusion or extrusion and reinforced with 3 wt.% coconut or sisal fibers, together with an unreinforced reference and selected brushed-surface variants, were evaluated after 12 months in soil or water. Mass change and three-point flexural properties were quantified using five specimens per condition, and the culturable surface communities were characterized by 16S rRNA (V3–V4) and ITS1 amplicon sequencing. Mass gain remained low (0.116–0.580%), although water exposure and surface brushing significantly increased uptake. Across all material-exposure variants, flexural strain at break, flexural stress at break, flexural modulus, and maximum flexural stress decreased by only 0.3–1.1%, and every uncertainty-normalized shift remained below 0.5 propagated standard errors. Bacterial communities were dominated by *Pseudomonas*, *Serratia*, or *Bacillus*, whereas fungal profiles separated into *Naganishia*/*Filobasidium*-rich and *Mucor*-dominated assemblages. A literature-based trait analysis revealed high cumulative abundances of genera with documented cellulose- and ester-hydrolyzing capacity and, among bacteria, polyethylene/alkane-oxidizing capacity. The results indicate persistent biological colonization of the composite surfaces, whereas bulk flexural performance remained essentially unchanged after one year of environmental exposure.

## 1. Introduction

Plant-fiber-reinforced thermoplastic composites are increasingly considered for semi-structural and building applications because they combine low density, recoverable polymer matrices, and renewable or waste-derived reinforcement. Their engineering value depends on their durability under prolonged exposure to moisture, which induces swelling of lignocellulosic fibers, modifies the fiber-matrix interphase, and alters stiffness and strength; these mechanisms are recognized as central durability issues for natural-fiber composites [1]. A recent review focusing specifically on construction biocomposites identified moisture as the principal link between water uptake, fungal activity, and interfacial deterioration [2].

Polyethylene matrices are attractive in this context because of their chemical resistance and established processing routes. Published studies of natural-fiber/polyolefin composites report higher stiffness together with increased water uptake as hydrophilic reinforcement is introduced into the matrix [3,4]. Bazan et al. showed that natural fibers in polyethylene alter both the mechanical response and water uptake and that water-aged formulations retain substantial mechanical performance when the matrix-filler architecture remains stable [3]. A recent study on low-density polyethylene (LDPE)/alfa-fiber composites further demonstrated moisture-driven interfacial debonding and measurable mechanical losses at high fiber content and saturation [5].

Recycled textiles provide an additional circular-economy route. Mixed textile waste contains polymeric and biopolymeric components suitable for reprocessing as reinforcement or filler instead of landfill disposal. This route has become particularly relevant since separate collection of textile waste became mandatory in the European Union on 1 January 2025 [6]. However, fiber-to-fiber recycling of blended polyester/cellulose textiles remains technically limited, even though the reuse and recycling of textiles provide clear environmental benefits over incineration and landfilling [7]. Echeverria et al. reported moisture-resistant textile-fiber composites for load-bearing and non-load-bearing building applications [8], while Kamble and Behera developed textile-waste hybrid composites with competitive strength and modulus for construction products [9]. Recent work by Lipka et al. on related recycled textile-plant fiber composites showed that extrusion processing with 3–6 wt.% cellulose-based plant reinforcement produced distinct mechanical, structural, and morphological responses across fiber types [10]. Together, these studies document the engineering applicability of such material systems; however, long-term datasets coupling mechanical durability with microbial colonization remain scarce. The present study extends this material platform to one-year environmental durability and microbial colonization.

Microbial colonization adds a second durability dimension. Plastic surfaces rapidly develop specific microbial communities, often described as the plastisphere [11]. In plant-filled polyolefin systems, the cellulosic fraction provides an accessible carbon source and a pathway for moisture ingress. Soil-burial studies of LDPE composites containing lignocellulosic fillers have reported progressive mass and strength losses over longer exposure periods [12]. Plastisphere succession studies also report enrichment of hydrocarbon-degrading taxa in polymer-associated bacterial communities [13]. However, surface colonization does not necessarily imply bulk polymer failure; the engineering question is whether the biological layer is accompanied by a measurable loss of load-bearing performance.

Compared with earlier work, the novelty of the present study lies in four aspects. First, most durability data for natural-fiber composites, including polyolefin-based systems, have been obtained by accelerated or saturation-based aging (immersion, often at elevated temperature, or hygrothermal cycling) of small laboratory coupons [3,5,14,15], and such tests may overestimate property losses relative to service conditions; real-time exposure data in soil and natural waters remain scarce, and their lack has been identified as a major barrier to the acceptance of natural-fiber composites in infrastructure [16,17]. Second, the matrix system studied here is not a virgin polyolefin with a single fiber type [3,4,5,18], but a recycled, multicomponent material in which LDPE binds mixed post-consumer textile waste (polyester and cellulosic fibers) and a small addition (3 wt.%) of plant fibers. Third, earlier soil-burial studies of LDPE/lignocellulosic composites quantified mass and strength losses [12] but did not identify the colonizing microorganisms, whereas plastisphere studies [11,13] have mostly addressed films and debris rather than engineering composites with measured mechanical properties. Fourth, the tested specimens were product-scale elements (approximately 1.2–1.5 kg each), so that the surface-to-volume ratio and the processing-induced surface skin were representative of real building products.

From a structural standpoint, the investigated composites are multiphase systems whose durability is expected to be governed by four features: (i) the continuous, nonpolar, semicrystalline LDPE matrix, which has very low water solubility and encapsulates the other phases; (ii) the polyester (PET) fibers of the textile fraction, which do not melt at the processing temperatures used (170–200 °C; Section 2.1) and therefore remain a fibrous, weakly hygroscopic reinforcement; (iii) the hygroscopic cellulosic phases (cotton/viscose-type textile fibers and coir or sisal), whose hydroxyl groups, hemicellulose, and lumen porosity provide moisture-uptake sites and an accessible carbon source for microorganisms [17,19]; and (iv) the fiber-matrix interface, which lacks chemical bonding and can act as a capillary pathway. The study design therefore relates the measured gravimetric, mechanical, and microbiological responses to these structural features.

The present work links three complementary data streams after 12 months of soil or water exposure: gravimetric response, three-point flexural performance, and culture-based 16S rRNA/ITS1 community profiling. The study focuses on recycled textile-LDPE composites produced by two processing routes and modified with 3 wt.% coconut or sisal fibers, with selected brushed-surface variants. The objective was to determine whether one year of environmental exposure leads to coupled changes in mass, flexural response, and microbial community structure. Specifically, the study tested whether encapsulation of the hygroscopic cellulosic phases in the continuous hydrophobic LDPE matrix restricts moisture ingress and microbial access to the near-surface layer, and thereby decouples surface colonization from the bulk flexural response. The central finding is a clear separation between surface biological activity and bulk mechanical response: viable, functionally diverse communities were recovered from the surfaces, while flexural properties changed by no more than 1.1%.

The paper is organized as follows. Section 2 describes the raw materials, the rationale for their selection, the intended application, and the test methods. Section 3 presents the results in two blocks: the bulk material response (mass change and flexural properties; Section 3.1 and Section 3.2) and the surface microbiology (sequencing performance, community composition, diversity, and functional profile; Section 3.3, Section 3.4, Section 3.5, Section 3.6, Section 3.7 and Section 3.8). Section 4 interprets the mass uptake in terms of porosity and surface chemistry (Section 4.1), the separation of surface colonization from bulk response (Section 4.2), the functional ecology of the recovered communities (Section 4.3), and the structure-property relationships of the composites (Section 4.4); it then discusses practical implications for the intended applications (Section 4.5) and the limitations of the study (Section 4.6). Section 5 summarizes the main findings.

## 2. Materials and Methods

### 2.1. Composite Variants and Environmental Exposure

The investigated materials were recycled textile-LDPE composites containing a heterogeneous textile fraction with cellulosic components (including cotton/viscose-type fibers) and a polyester component accounting for approximately 58% of the textile fraction. Three material families were included in the experimental dataset: an intrusion-processed composite reinforced with 3 wt.% coconut fibers, an extrusion-processed composite reinforced with 3 wt.% sisal fibers, and an intrusion-processed reference without additional plant-fiber reinforcement. Selected coconut and sisal variants were mechanically brushed before exposure to increase surface accessibility. The broader environmental incubation protocol covered freshwater, saline water, and multiple soil types over 12 months. For the gravimetric and flexural analyses presented here, the specimen coding groups the environments as water (W) or soil (G). The specimen coding used throughout the study is summarized in Table 1.

The microbial libraries followed an independent coding scheme: Y-1, Y-3, Y-4, and Y-6 for bacteria and S-4, S-5, S-6, and S-7 for fungi. These libraries represent the broader incubation set spanning freshwater, saline water, and multiple soil types. Accordingly, the microbiological results are reported at the library level and are not cross-assigned to the mechanical specimen codes.

**Raw materials and processing.** The textile fraction was supplied by VIVE Textile Recycling (Poland) as shredded mixed post-consumer textile waste consisting of approximately 58% polyester, 25% cotton/viscose-type cellulosic fibers, 4.5% nylon, and 12.5% other fibers, including elastane (spandex), viscose, and lyocell. The LDPE was recovered from the post-consumer polyethylene bags in which used clothing is collected for recycling; the ground LDPE had a melt flow index (MFI) of 0.9 g/10 min (190 °C/2.16 kg). Because the molecular weight of LDPE in a recycled multicomponent compound cannot be determined directly by high-temperature gel permeation chromatography (GPC) without prior separation of the phases, the MFI is reported as a processing-relevant indicator of the molecular weight of the matrix. The textile and plant components were comminuted to fragments of approximately 2–15 mm and compounded with LDPE. As in the preceding study on the same material platform [10], two processing routes were used: intrusion (low-pressure filling of a mold with the plasticized mixture) and extrusion. The test specimens were produced by extruding the mixtures into molds with a single-screw extruder at processing temperatures of 170–200 °C (zone 1, 170 °C; zone 2, 175 °C; zone 3, 175 °C; zone 4, 200 °C; zone 5, 195 °C; zone 6, 190 °C; residence time, 5 min; screw speed, 70 rpm); the molds were kept at 20 °C without external cooling. At these temperatures, LDPE is fully molten, whereas the PET and cellulosic fibers remain solid; the processing history of the fibrous phases is therefore limited to one service life and one thermomechanical compounding step.

**Choice of textile-based reinforcement.** Mixed textile waste was selected as the principal reinforcing phase for three reasons. First, it is an abundant waste stream with limited recycling outlets: separate collection of textiles has been mandatory in the EU since 1 January 2025 [6], whereas fiber-to-fiber recycling of polyester/cellulose blends is technically difficult, and most collected material is still downcycled, incinerated, or landfilled [7]. Second, its intrinsic properties suit a thermoplastic composite: the polyester fraction (approximately 58%) does not melt during LDPE processing and acts as a stiff, weakly hygroscopic fibrous reinforcement, whereas the cotton/viscose fraction contributes additional stiffness at low density. Third, the composite route accepts the heterogeneous fiber composition of post-consumer textiles without costly sorting by fiber type, which allows a high share of waste-derived material in the final product. The plant fibers were therefore used only as a 3 wt.% secondary modifier of an already fiber-rich recycled system.

**Selection of plant fibers.** Coir and sisal were selected on the basis of the preceding screening of eight plant fibers (sisal, cotton, flax, coconut, wood, abaca, jute, and hemp) incorporated at 3–6 wt.% into the same recycled textile platform [10]. In that screening, 3 wt.% sisal provided a flexural modulus and maximum flexural stress comparable to those of the glass-fiber reference. Coir was retained because of its distinctly different chemistry: with approximately 40–45% lignin and 32–43% cellulose, it is one of the most lignin-rich plant fibers, whereas sisal (approximately 65% cellulose and 10% lignin) and bast fibers such as flax (approximately 71% cellulose and 2% lignin) are cellulose-rich [19]. The two selected fibers therefore represent the two ends of the lignin/cellulose range that is relevant to moisture sensitivity and microbial accessibility, because lignin is hydrophobic and comparatively resistant to microbial attack, whereas cellulose and hemicellulose provide hydroxyl groups and readily accessible carbon [17]. Bast fibers cultivated in Central Europe (flax and hemp) were not excluded for technical reasons (they were included in the screening [10]); rather, they are mainly directed to higher-value textile and technical-composite markets [20], and their low-lignin, cellulose-rich composition largely duplicates that of the cotton/viscose fraction already present in the textile waste. Coir and sisal, by contrast, are available on the European market as low-cost industrial fibers. Long-term exposure of composites with regionally cultivated bast fibers is a logical continuation of this work.

**Fiber preparation and inorganic impurities.** The plant fibers were used in their native state, without alkali treatment, bleaching, or other chemical purification, and were only mechanically comminuted before compounding [10]. Before mixing, the fibers were conditioned for 2 days at 20 ± 2 °C and 40–60% relative humidity, and they were mixed with the textile granulate under the same conditions. The use of untreated fibers was deliberate. Chemical purification removes hemicellulose, pectin, waxes, and part of the lignin, enriches the fibers in alpha-cellulose, and changes their supramolecular structure and surface morphology, as shown for flax and hemp bast fibers [21] and reviewed for alkali, silane, and other treatments [22]. In polar matrices such treatments usually improve interfacial adhesion; in a nonpolar LDPE matrix without a coupling agent, however, the dominant effect of impurity removal would be an increase in accessible hydroxyl groups and surface roughness, i.e., higher hygroscopicity and greater availability of polysaccharides to microorganisms [17,22]. The native waxes and lignin, in contrast, form a hydrophobic and less biodegradable sheath around the cellulose microfibrils; untreated fibers also avoid the chemical consumption and effluents of purification, in line with the circular-economy objective. The ash and metal contents of the fibers were not determined in this study. Even assuming an ash content as high as 5 wt.% in the native fibers, the plant-fiber contribution to the inorganic content of the composite at 3 wt.% loading would not exceed 0.15 wt.%. Transition-metal ions (e.g., Fe, Mn, Co) are known pro-oxidants for polyethylene that can accelerate the abiotic oxidation preceding biodegradation [23]; at the trace levels expected here, and without UV irradiation during soil or water exposure, no such effect was evident in the flexural data.

**Intended application and service conditions.** The composites are intended for non-load-bearing and semi-structural outdoor building and landscaping elements manufactured as boards and profiles, such as lawn and garden edging, palisades, raised-bed and low retaining boards, fence boards, and terrace substructure elements. In service, these elements are partly embedded in soil or periodically wetted or immersed in fresh or saline water (e.g., at pond edges, in drainage channels, and in coastal areas), which is why these environments were included in the incubation protocol. The dominant mechanical action is bending: lateral earth pressure acting on partly embedded edging and retaining boards, and spans loaded by self-weight, wind, or foot traffic in fence and terrace elements. Local contact stresses (indentation by stones or tools) and impact during installation are secondary actions. Three-point flexural testing was therefore selected as the primary mechanical criterion, in line with the test methods specified for natural-fiber-thermoplastic composite products [24]; the additional properties required for full qualification are discussed in Section 4.6.

### 2.2. Mass-Change Measurements

Five replicate specimens were evaluated for each material-exposure variant. The mass of each specimen was recorded before exposure (*m*_0_) and after 12 months (*m*_12_). The absolute mass gain was calculated as Δ*m* = *m*_12_ − *m*_0_, and the relative mass gain as Δ*m* (%) = [(*m*_12_ − *m*_0_)/*m*_0_] × 100. Group values are reported as the mean ± standard deviation (SD). The raw replicate data were used for the inferential statistics described in Section 2.7.

### 2.3. Three-Point Flexural Testing

Three-point flexural testing was performed before environmental exposure and after 12 months using five specimens per condition and the same laboratory protocol at both time points. The reported quantities were flexural strain at break (ε_fb_, %), flexural stress at break (σ_fb_, MPa), flexural modulus (*E*_f_, MPa), and maximum flexural stress (σ_fM_, MPa). Results are reported as the mean, SD, and standard error of the mean (SEM). The relative change was calculated as (*x*_12_ − *x*_0_)/*x*_0_ × 100. To compare each before/after shift with its reported uncertainty, the propagated standard error was calculated as SE_Δ_ = √(SEM_0_^2^ + SEM_12_^2^), and the uncertainty-normalized shift was expressed as |Δ*x*|/SE_Δ_.

### 2.4. Recovery and Cultivation of Surface Microorganisms

Material collected from the composite surfaces after 12 months was cultured on Plate Count Agar (PCA) for bacteria and Sabouraud Dextrose Agar (SDA) for fungi at 28 °C. DNA was isolated from the resulting biomass. Consequently, the taxonomic profiles describe the culturable and viable fraction of the communities that colonized the composite surfaces and retained the capacity to grow after long-term exposure.

### 2.5. 16S rRNA and ITS1 Amplicon Processing

Four bacterial libraries (Y-1, Y-3, Y-4, Y-6) targeted the V3–V4 region of the 16S rRNA gene. Reads were processed in QIIME 2 [25] using FIGARO (https://github.com/Zymo-Research/figaro, accessed on 26 October 2026) [26], Cutadapt [27], and DADA2 (https://benjjneb.github.io/dada2/, accessed on 26 October 2026) [28]. Amplicon sequence variants were classified against SILVA 138 [29] using VSEARCH (https://github.com/torognes/vsearch, accessed on 26 October 2026) [30] and the q2-feature-classifier/scikit-learn workflow [31]. Multiple-sequence alignment and phylogenetic tree construction were performed with MAFFT v7 [32] and FastTree 2 [33]. The 341F/785R primer pair corresponded to the broadly validated V3–V4 primer set described by Klindworth et al. [34].

Four fungal libraries (S-4, S-5, S-6, S-7) targeted ITS1. The workflow used QIIME [35], Cutadapt, fastp v0.23.4 [36], usearch61/UCLUST (USEARCH v6.1) [37], and BLAST (https://blast.ncbi.nlm.nih.gov/, accessed on 26 October 2026)-based taxonomic assignment against UNITE v8.2 [38]. Relative abundance was calculated as the percentage of classified reads. Taxa outside the list of dominant taxa were pooled as “Other taxa”, and unassigned reads were retained as “Unidentified”.

### 2.6. Diversity Metrics and Literature-Based Functional Profile

Genus-level alpha diversity was described using Shannon H’ [39], Simpson 1-D and inverse Simpson 1/D [40], Pielou evenness J [41], and Berger-Parker dominance d [42]. Genus-level beta diversity was quantified with Bray-Curtis dissimilarity [43], followed by UPGMA clustering and principal coordinates analysis (PCoA) [44].

The literature-based functional profile was calculated by assigning each recovered genus to literature-supported ecological traits: (hemi)cellulose degradation, ester/polyester hydrolase activity, polyethylene/alkane oxidation, chitin degradation, and biofilm/extracellular-polymeric-substance formation. The score for a trait was the sum of the relative abundances of genera for which that trait has been documented. This metric represents a community-level trait profile derived from documented genus-level functions and does not represent directly measured enzyme activity. The interpretation was based on the literature describing plastic-associated communities and polymer-degrading taxa [11,13,45,46,47,48,49,50].

### 2.7. Statistical Analysis

Mass-gain statistics were calculated from the five raw replicate measurements per group. For the coconut and sisal material families, two-way analysis of variance (ANOVA) tested the effects of environment (soil versus water), surface state (unbrushed versus brushed), and their interaction. Partial eta-squared (η_p_^2^) was reported as a measure of effect size. For the unreinforced intrusion reference, soil and water groups were compared using Welch’s *t*-test. An overall one-way ANOVA across all ten material-exposure groups was used as a descriptive omnibus test. Significance was assessed at α = 0.05. Flexural data were analyzed through relative changes and the uncertainty-normalized shift defined in Section 2.3. Microbial community comparisons used compositional, alpha-diversity, and Bray-Curtis beta-diversity metrics rather than parametric group testing.

## 3. Results

The results are presented from the bulk material response to the surface microbiology. Section 3.1 and Section 3.2 report the gravimetric and flexural responses of the composite specimens; Section 3.3 reports the sequencing performance; Section 3.4, Section 3.5, Section 3.6 and Section 3.7 describe the composition and diversity of the recovered communities; and Section 3.8 presents their literature-based functional profile.

### 3.1. Mass Uptake After 12 Months

**Figure 1:**
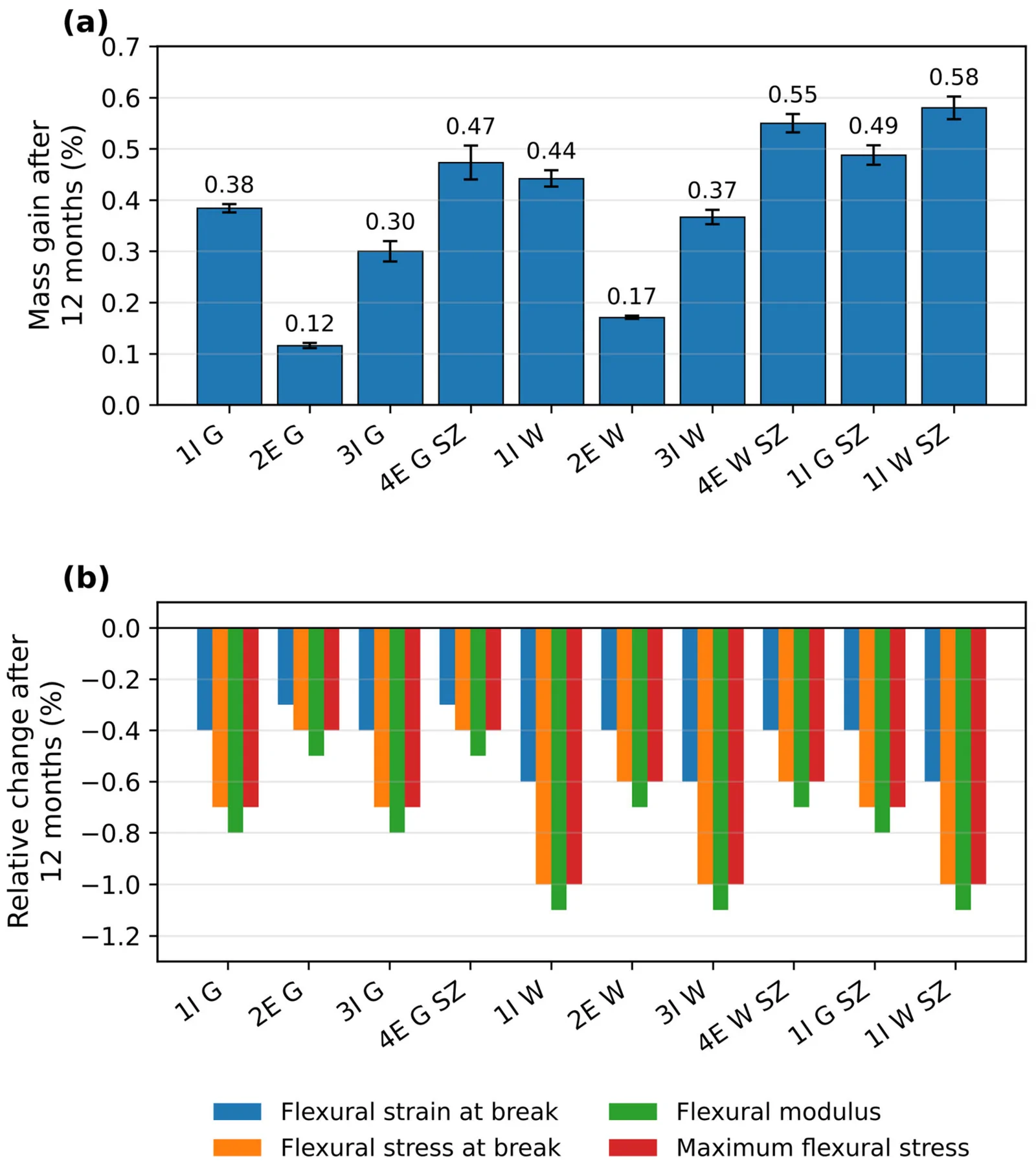
Macroscopic durability after 12 months. (**a**) Relative mass gain (mean ± SD, *n* = 5). (**b**) Relative changes in flexural strain, flexural stress at break, flexural modulus, and maximum flexural stress.

All groups showed a small positive mass change rather than a net mass loss (Table 2; Figure 1a). Mean relative gain ranged from 0.116 ± 0.005% for 2E G to 0.580 ± 0.022% for 1I W SZ. Water exposure increased mass gain in every directly matched material pair. Brushing produced the largest increase, particularly for the sisal/extrusion material: the unbrushed variants gained 0.116–0.171%, whereas the brushed variants gained 0.473–0.550%.

Two-way ANOVA revealed strong effects of both exposure medium and surface state. In the coconut family, the environment effect was *F*(1,16) = 95.23, *p* = 3.85 × 10^−8^, η_p_^2^ = 0.856, and the brushing effect was *F*(1,16) = 248.54, *p* = 3.62 × 10^−11^, η_p_^2^ = 0.940. The interaction was also significant, *F*(1,16) = 5.16, *p* = 0.037, η_p_^2^ = 0.244. In the sisal family, environment gave *F*(1,16) = 59.64, *p* = 8.73 × 10^−7^, η_p_^2^ = 0.788, and brushing gave *F*(1,16) = 1875.44, *p* = 5.17 × 10^−18^, η_p_^2^ = 0.992; their interaction was not significant (*p* = 0.220). The unreinforced reference also gained more mass in water than in soil (0.367% vs. 0.300%; Welch’s *t* = −6.20, df = 7.15, *p* = 4.08 × 10^−4^). Across all ten groups, the omnibus ANOVA yielded *F*(9,40) = 369.15, *p* = 1.38 × 10^−35^, η^2^ = 0.988.

### 3.2. Flexural Stability

The flexural response remained highly stable over the 12-month exposure (Figure 1b; Table 3). Relative changes were negative in all reported parameters, but their magnitude was small: flexural strain at break changed by −0.3 to −0.6%, flexural stress at break by −0.4 to −1.0%, flexural modulus by −0.5 to −1.1%, and maximum flexural stress by −0.4 to −1.0%. Every uncertainty-normalized shift remained below 0.50 SE_Δ_, including the largest values, obtained for σ_fb_ in 2E W and 4E W SZ (0.48) and for *E*_f_ in 3I W (0.43).

The processing route and reinforcement controlled the absolute flexural response more strongly than the 12-month exposure. The extrusion/sisal groups exhibited the highest flexural modulus (approximately 2.00–2.02 GPa) and a low flexural strain at break (approximately 1.14%), whereas the intrusion-processed coconut variants showed a larger strain at break (approximately 6.3%) and a maximum flexural stress of approximately 24 MPa. These material-specific signatures were retained after exposure.

### 3.3. Sequencing and Classification Performance

**Figure 2:**
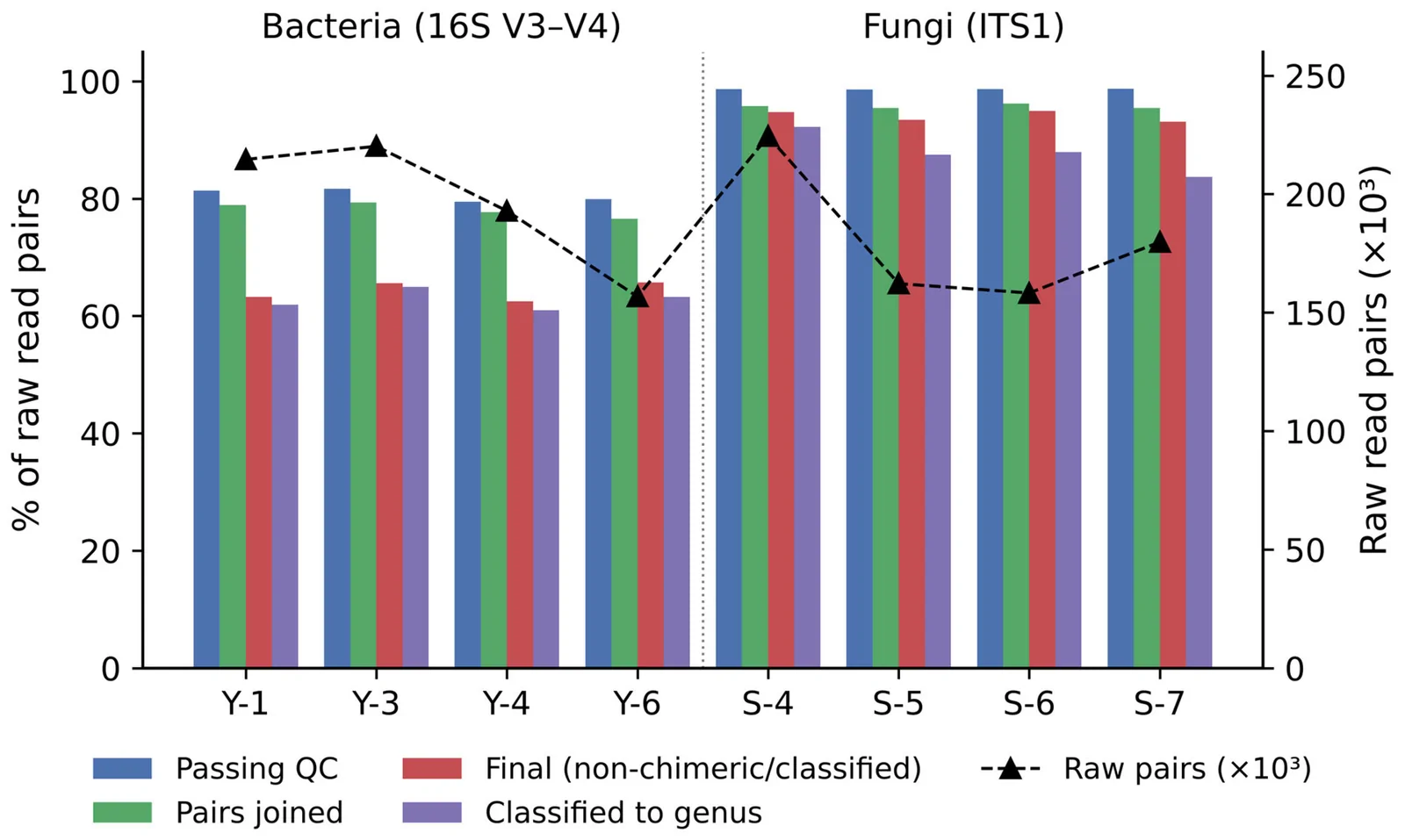
Sequencing and classification performance of 16S rRNA (V3–V4) and ITS1 libraries recovered from composite surfaces after 12-month environmental incubation. Bars show the percentage of raw read pairs retained at each step; triangles show raw read-pair counts.

The amplicon libraries contained 156,724–224,408 raw read pairs (Table 4; Figure 2). ITS1 libraries retained 98.59–98.72% of pairs after quality filtering and 93.09–94.97% as final classified reads. For the 16S V3–V4 libraries, DADA2 processing yielded 62.52–65.73% final non-chimeric reads, corresponding to more than 100,000 reads per library. Genus-level classification was high for both markers: 96.24–99.05% for bacteria and 83.72–92.25% for fungi. Species-level resolution was markedly higher for ITS1 (76.49–90.94%) than for 16S (9.65–44.52%), and the bacterial interpretation therefore focuses on genus-level patterns.

### 3.4. Bacterial Community Structure

**Figure 3:**
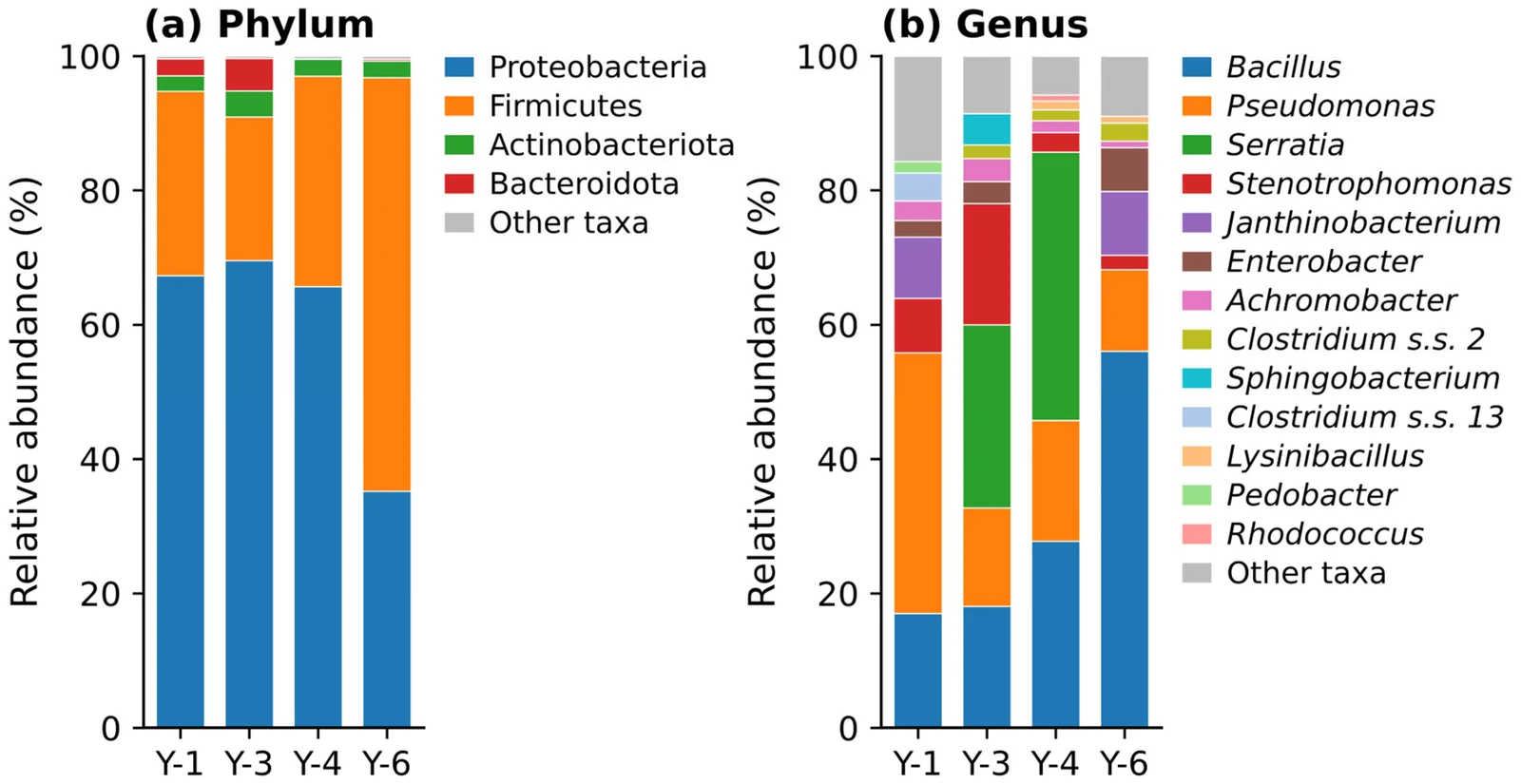
Relative abundance of culturable bacteria recovered from composite surfaces at the (**a**) phylum and (**b**) genus levels (16S rRNA V3–V4).

All bacterial libraries contained the same four major phyla: Proteobacteria, Firmicutes, Actinobacteriota, and Bacteroidota (Figure 3a). Proteobacteria dominated Y-1, Y-3, and Y-4 (65.66–69.57%), whereas Y-6 shifted strongly toward Firmicutes (61.61%). At the genus level, Y-1 was dominated by *Pseudomonas* (38.84%), Y-3 and Y-4 by *Serratia* (27.27% and 40.00%), and Y-6 by *Bacillus* (56.05%) (Figure 3b). *Stenotrophomonas* reached 18.09% in Y-3, while *Janthinobacterium* contributed approximately 9% in Y-1 and Y-6. *Bacillus*, *Pseudomonas*, *Stenotrophomonas*, *Achromobacter*, and *Clostridium* sensu stricto formed a recurring bacterial core across the four libraries.

### 3.5. Fungal Community Structure

**Figure 4:**
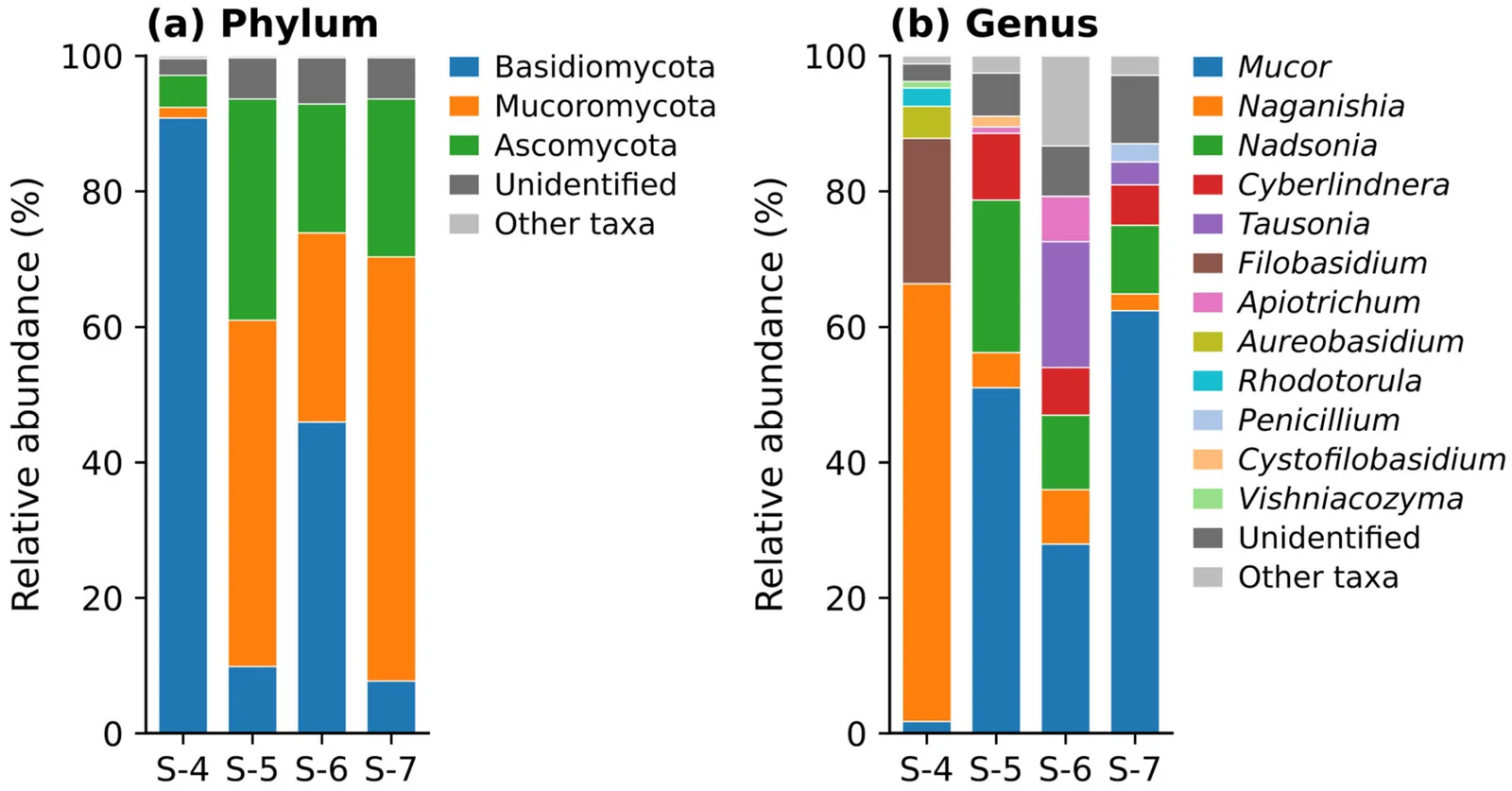
Relative abundance of culturable fungi recovered from composite surfaces at the (**a**) phylum and (**b**) genus levels (ITS1).

Fungal communities separated into two distinct compositional patterns (Figure 4). S-4 consisted primarily of Basidiomycota (90.81%), especially *Naganishia* (64.67%) and *Filobasidium* (21.47%), with smaller contributions from *Aureobasidium* (4.71%) and *Rhodotorula* (2.71%). In contrast, S-5, S-6, and S-7 shared a *Mucor*/*Nadsonia*/*Cyberlindnera* framework. *Mucor* reached 51.00% in S-5 and 62.37% in S-7; S-6 was more even, with *Mucor* (27.91%), *Tausonia* (18.62%), *Nadsonia* (10.98%), *Naganishia* (8.09%), *Cyberlindnera* (7.00%), and *Apiotrichum* (6.69%).

### 3.6. Species-Level Patterns

**Figure 5:**
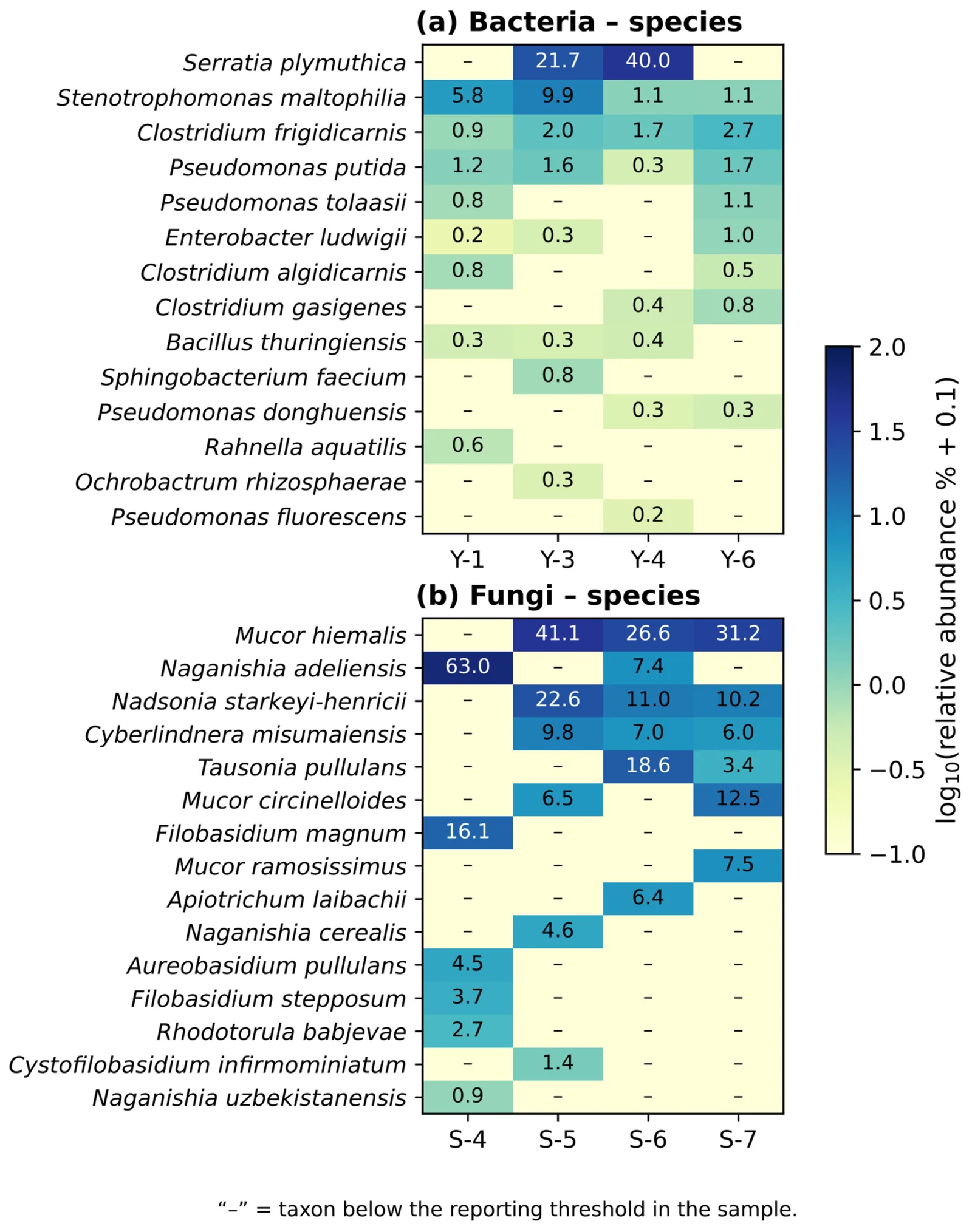
Heat map of dominant bacterial (**a**) and fungal (**b**) species. Values are relative abundances (% of classified reads); the color scale is log_10_(% + 0.1). White or black numbers in the cells are used only to ensure legibility against the cell color.

Species-level assignments refined the ecological interpretation (Figure 5). *Serratia plymuthica* accounted for 21.71% of Y-3 and 40.00% of Y-4. *Stenotrophomonas maltophilia* occurred in all four bacterial libraries, with a maximum of 9.91% in Y-3. *Pseudomonas putida* was consistently present at lower abundance (0.32–1.68%). Psychrotolerant *Clostridium* species, including *C. frigidicarnis*, *C. algidicarnis*, and *C. gasigenes*, were also detected.

Among fungi, *Mucor hiemalis* dominated S-5 (41.13%) and remained abundant in S-6 (26.62%) and S-7 (31.19%). S-7 additionally contained *M. circinelloides* (12.45%) and *M. ramosissimus* (7.53%). S-4 was instead characterized by *Naganishia adeliensis* (63.00%), *Filobasidium magnum* (16.13%), and *Aureobasidium pullulans* (4.54%). *Nadsonia starkeyi-henricii* and *Cyberlindnera misumaiensis* were persistent components of S-5 to S-7.

### 3.7. Alpha and Beta Diversity

**Figure 6:**
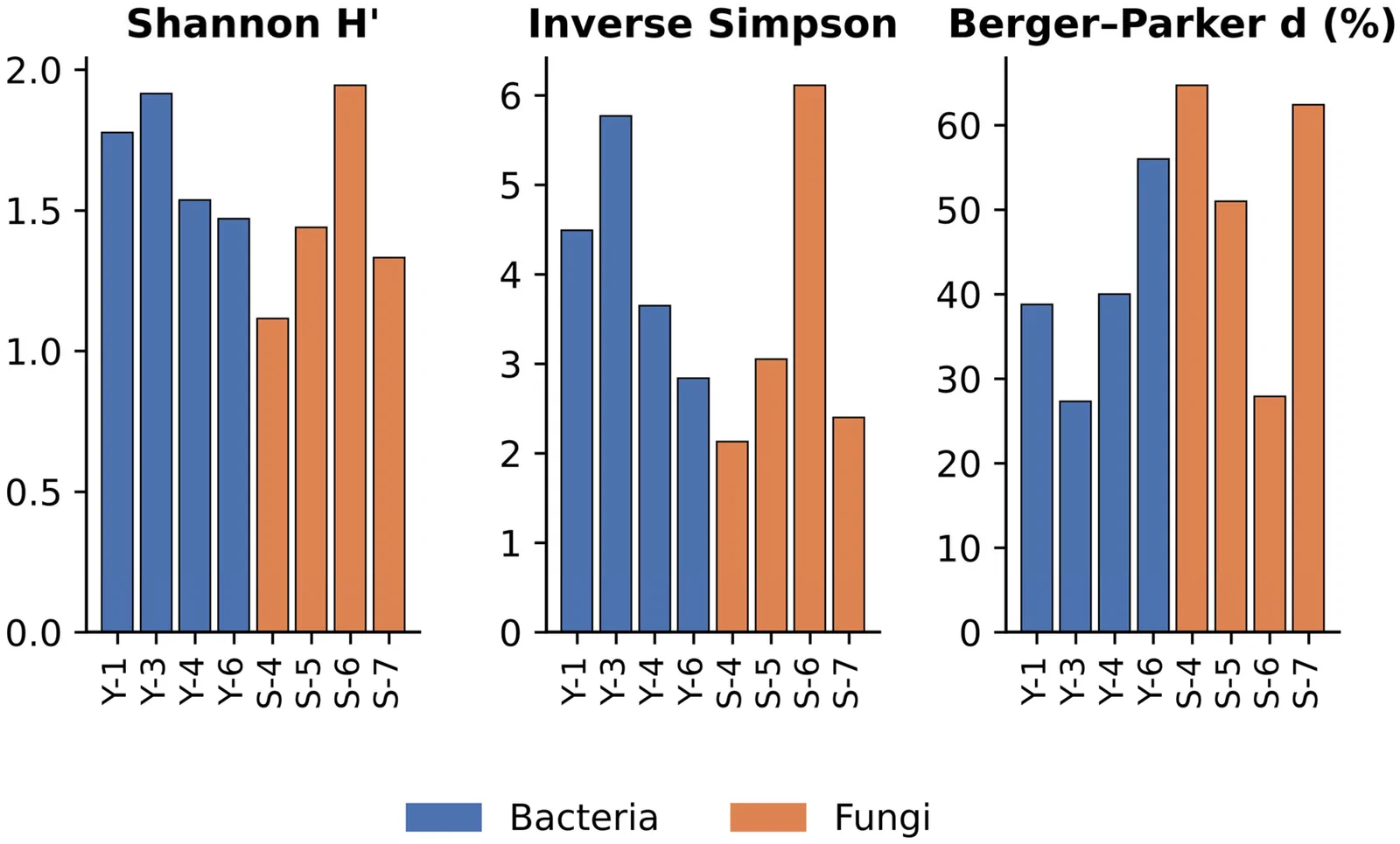
Genus-level alpha-diversity indices of bacterial and fungal communities: Shannon H’, inverse Simpson, and Berger-Parker dominance.

Bacterial Shannon diversity ranged from H’ = 1.470 to 1.914, with Y-3 being the most even community (J = 0.871) and Y-6 the most strongly dominated (Berger-Parker d = 56.0%). Fungal diversity varied more widely. S-6 had the highest diversity in the full dataset (H’ = 1.944; inverse Simpson = 6.11; J = 0.935), whereas S-4 and S-7 were strongly dominated by *Naganishia* and *Mucor*, respectively (Table 5; Figure 6).

**Figure 7:**
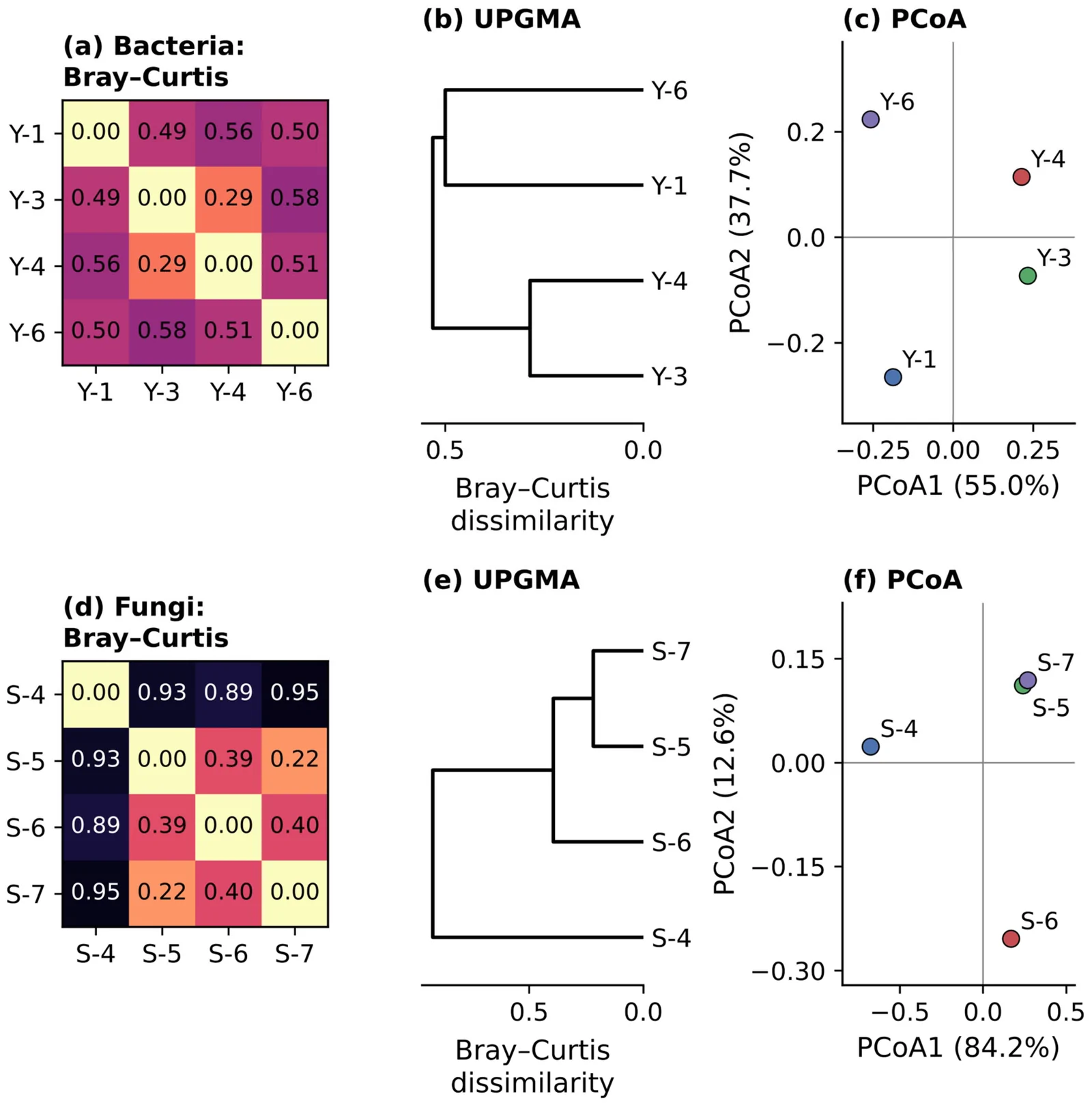
Beta diversity of bacterial (**a**–**c**) and fungal (**d**–**f**) communities: Bray-Curtis dissimilarity matrices, UPGMA clustering, and principal coordinates analysis at genus level. In (**a**,**d**), white or black numbers are used only to ensure legibility against the cell color; in (**c**,**f**), marker colors only distinguish the individual libraries, which are also labeled.

Bray-Curtis analysis showed that Y-3 and Y-4 were the closest bacterial pair (0.286), consistent with shared *Serratia* dominance. Bacterial dissimilarities otherwise ranged from 0.487 to 0.577. The first two bacterial PCoA axes explained 92.7% of the variance (55.0% and 37.6%). Fungal communities were more clearly separated: S-4 differed from S-5/S-6/S-7 by 0.888–0.954, whereas S-5 and S-7 were highly similar (0.219). The first fungal PCoA axis alone explained 84.2% of the variance, separating the Filobasidiales-rich S-4 profile from the *Mucor*-rich group (Figure 7).

### 3.8. Literature-Based Functional Profile

**Figure 8:**
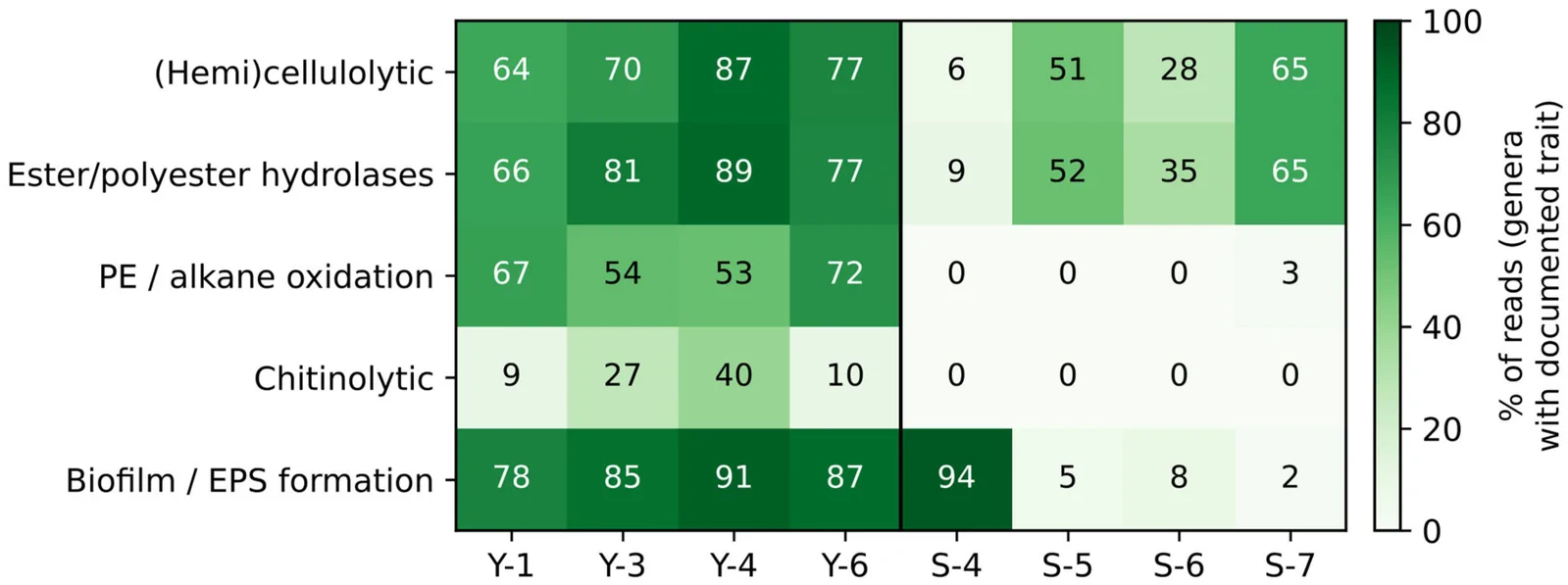
Literature-based functional profile of recovered communities: cumulative relative abundance (%) of genera associated with (hemi)cellulolysis, ester/polyester hydrolases, polyethylene/alkane oxidation, chitinolysis, and biofilm/EPS formation.

The bacterial libraries showed high cumulative abundances of genera associated with (hemi)cellulose degradation (64.2–87.4%), ester/polyester hydrolysis (66.4–88.6%), and biofilm/EPS formation (78.4–91.3%) (Table 6; Figure 8). The trait-assigned cumulative abundance for polyethylene/alkane oxidation ranged from 52.6% to 72.3%. The chitinolytic trait score was highest in the *Serratia*-rich Y-4 library (40.0%). Fungal functional profiles were more polarized: S-5 and S-7 showed the highest cumulative abundances of genera associated with (hemi)cellulolytic and ester-hydrolytic functions, whereas S-4 was dominated by biofilm/EPS-forming yeasts (93.6%).

## 4. Discussion

### 4.1. Small Mass Gain and High Flexural Retention

The macroscopic response after one year is characterized by a small positive mass gain with almost unchanged flexural properties. This combination is noteworthy because moisture-sensitive natural-fiber composites commonly show stronger coupling between uptake and mechanical deterioration. Reviews of plant-fiber composite durability identify moisture-driven fiber swelling, matrix plasticization, and interfacial weakening as the dominant causes of property loss [1,2]. In natural-fiber/polyolefin composites, water uptake generally increases with the accessible hydrophilic fraction [3,4]. Nevertheless, the present values are low: the mass gain of all groups remained below 0.60% after 12 months.

Surface brushing had a larger effect on mass gain than the change from soil to water, particularly for the sisal/extrusion material. For this material, partial eta-squared reached 0.992 for brushing, compared with 0.788 for the environment. The effect-size comparison identifies surface state as the dominant factor controlling liquid or residue retention in these composites. The mechanically opened surface increased the accessibility of the textile and plant-fiber phases, consistent with the measured increase in mass gain. The significant interaction in the coconut family further shows that the water effect was amplified after brushing.

The low uptake values can be explained by the porosity and surface chemistry of the composites. In plant-fiber-reinforced polymer composites, water is transported by three mechanisms: diffusion through the matrix (negligible for nonpolar LDPE), capillary flow along fiber-matrix interfacial gaps, fiber lumens, and microvoids, and transport along microcracks [15,17]. The amount of absorbed water is therefore controlled by the number of accessible hydroxyl groups of cellulose and hemicellulose and by the volume of open, surface-connected porosity, rather than by the nominal fiber content alone [17,19]. In the present composites, the hygroscopic phases are encapsulated in a continuous LDPE phase that forms a polymer-rich skin during intrusion or extrusion, and the product-scale specimens (approximately 1.2–1.5 kg, back-calculated from Table 2) had a low ratio of exposed surface to volume. The data support this interpretation. The extrusion-processed sisal composite, which is the most highly consolidated, showed the lowest uptake (0.116–0.171%), whereas removal of the surface skin by brushing increased uptake of the same formulation three- to four-fold (0.473–0.550%). For the intrusion-processed coconut composite, brushing increased uptake by only about 30% (from 0.384 to 0.488% in soil and from 0.442 to 0.580% in water), indicating a thinner or more porous skin already in the as-processed state. Because brushing changes only the surface, this response identifies open surface porosity and exposed, hydroxyl-rich fiber ends, rather than the bulk formulation, as the factors controlling moisture uptake. The moisture front thus remains confined to the near-surface layer. This is particularly relevant for flexural loading, in which the maximum normal stresses act in the outer layers; the absence of measurable flexural loss therefore indicates that the near-surface layer was not mechanically weakened to a detectable extent within 12 months. Part of the measured gain may also be attributed to adhering soil particles and biofilm biomass, which is consistent with the established surface colonization described in Section 4.2.

Despite this uptake, the mechanical signature of each material family was preserved. The maximum relative reduction across four flexural metrics was only 1.1%, and no shift exceeded 0.5 propagated standard errors. This contrasts with accelerated or saturation-based aging studies that reported losses of tens of percent in flexural properties. Lu and Van Vuure reported reductions of 14–48% in flexural strength and 6–57% in flexural modulus for flax-fiber thermoset composites, depending on the aging route [14]. Jellid et al. found a 23% strength decrease and moisture-driven interfacial debonding in saturated short alfa fiber/LDPE composites [5]. The considerably smaller changes observed here indicate retention of bulk load-transfer capacity in the tested recycled textile-LDPE composite architecture over the first annual exposure cycle.

### 4.2. Surface Colonization Without Detectable Bulk Flexural Damage

The microbiological results show that mechanical stability did not arise from a sterile or biologically inactive surface. Viable bacteria and fungi were recovered after 12 months, and the communities were taxonomically structured rather than random. This is consistent with the plastisphere concept, in which polymer surfaces act as selective ecological niches [11]. Wang et al. likewise reported an ordered succession of polyethylene-associated bacterial communities, together with the enrichment of taxa with hydrocarbon-degrading capability [13].

The environmental incubation included freshwater, saline water, and multiple soil types. Viable microbial communities were recovered across this heterogeneous exposure set, and the bacterial and fungal libraries retained recurrent dominant genera and clearly structured community profiles. The major microbiome signatures therefore remained evident across the environmental incubation matrix. Because the microbiological libraries used an independent coding scheme, taxonomic patterns are reported at the library level rather than assigned to individual water or soil subtypes.

The present data distinguish surface colonization from bulk degradation. Colonization was directly demonstrated by cultivation and amplicon sequencing, whereas bulk flexural degradation was not detected after 12 months. The positive gravimetric response coincided with established surface colonization and retained mechanical stability. Thus, the one-year exposure produced measurable surface biological activity without a measurable reduction in bulk flexural performance.

Longer soil-burial studies document a transition from surface colonization to material degradation under more severe exposure conditions. Zykova et al. reported 13–31% degradation after 20 months for LDPE films containing 30 wt.% wood flour or flax straw, together with mechanical losses and spectroscopic evidence of polymer-chain changes [12]. The higher lignocellulosic loading and longer exposure in that study resulted in a substantially more biodegradable system. The present one-year response instead represents a mechanically stable state in which colonization is established without major bulk deterioration.

### 4.3. Functional Ecology of the Recovered Communities

The bacterial profiles contained several genera repeatedly associated with polymer-surface colonization and hydrocarbon transformation. *Pseudomonas*, *Stenotrophomonas*, *Achromobacter*, and *Bacillus* contributed strongly to the trait-based polyethylene/alkane-oxidation score. Dey et al. reported LDPE degradation by *Stenotrophomonas* and *Achromobacter* isolates, including surface cracking and oxidative changes [45]. These literature-supported trait assignments indicate that these genera are relevant markers of the early material-biofilm interface, even when bulk polymer properties remain stable.

*Serratia plymuthica* was the defining species in Y-3 and Y-4. *Serratia* strains are well known for producing extracellular enzymes and for their antagonistic activity toward fungi [46], which is consistent with the elevated chitinolytic trait score of the *Serratia*-rich libraries. Y-6, in contrast, was dominated by *Bacillus* and formed a separate compositional endpoint. The bacterial beta-diversity analysis therefore reflects distinct ecological alternatives rather than a single uniform community.

The fungal data show an even stronger ecological split. S-4 was dominated by basidiomycetous yeasts, especially *Naganishia* and *Filobasidium*, and showed a high biofilm/EPS score and a low trait-assigned polysaccharide-hydrolysis score. S-5 to S-7 were progressively enriched in *Mucor*, including *M. hiemalis*, and contained higher cumulative abundances of genera associated with utilization of cellulosic or ester-containing components. Fungal attack on modified polyethylene-containing materials has been reported for *Mucor* and other fungi [47], while *Mucor* species are efficient producers of xylanolytic enzymes on lignocellulosic substrates [49]. The repeated detection of psychrotolerant yeasts such as *Naganishia* is also consistent with persistence across a full annual exposure cycle [48,50].

The functional heat map is a literature-based ecological trait map derived from documented genus-level functions. It identifies dominant genera associated with cellulose, polyester, or hydrocarbon transformation. The mechanically stable state observed here shows that a high trait score is not equivalent to a loss of engineering properties over the same exposure interval. The trait-based result therefore characterizes the documented functional profile of the material-surface community, whereas the flexural data independently quantify bulk mechanical durability.

### 4.4. Structure-Property Interpretation

The durability response can be rationalized by the structure of the composite. LDPE (melting range approximately 105–115 °C) forms the continuous matrix, whereas the PET fibers (melting point approximately 250–260 °C) and the cellulosic fibers do not melt at the processing temperatures used (170–200 °C; Section 2.1) and remain discrete fibrous reinforcements. Adhesion between the nonpolar LDPE and the polar PET and cellulosic surfaces is predominantly physical (wetting and mechanical interlocking) because no chemical coupling agent was used. The processing route controls fiber dispersion, orientation, and consolidation. The higher shear and pressure of extrusion favor fiber orientation along the flow direction and lower porosity, which explains the high flexural modulus (approximately 2.0 GPa) and low strain at break (approximately 1.1%) of the sisal/extrusion composites. The intrusion-processed materials, formed under lower shear, show a lower modulus (1.37–1.48 GPa) and a much larger strain at break (6.3–6.7%), i.e., a matrix-dominated, ductile response (Table A1). In an earlier study of the same material platform, scanning electron microscopy revealed a smooth and regular surface of the sisal composite, and FTIR confirmed the characteristic cellulose, hemicellulose, and lignin bands of the incorporated plant fibers [10].

These structural features also explain why surface colonization did not translate into bulk degradation. Enzymatic attack on high-molecular-weight polyethylene requires prior abiotic oxidation that introduces carbonyl groups and shortens the chains [23]; at ambient temperature and without UV irradiation, as during soil burial and immersion, this oxidation is very slow. Hydrolysis of PET ester bonds at ambient temperature is likewise slow. Consequently, although genera with documented polyethylene/alkane-oxidation and ester-hydrolase traits colonized the surfaces, the substrate effectively available to them within 12 months was mainly the cellulosic fraction exposed at the surface, together with adsorbed organic matter. The lignin-rich coir further limits the accessibility of cellulose to microorganisms. The structural interpretation therefore links the three data streams: a hydrophobic, encapsulating matrix limits moisture ingress (Section 3.1), the load-bearing cross-section remains intact (Section 3.2), and biological activity remains confined to the surface (Section 3.4, Section 3.5, Section 3.6, Section 3.7 and Section 3.8).

### 4.5. Implications for Construction-Oriented Recycled Composites

The combination of recycled textiles, LDPE, and plant fibers is relevant to boards, profiles, exterior accessories, fencing, edging, and other semi-structural products where moisture exposure and biological fouling occur over long service periods. Earlier textile-waste composites have already shown attractive moisture resistance and mechanical performance for building applications [8,9]. The present results add a time-dependent durability perspective: after one year, the composite family retains flexural performance despite established microbial colonization. These findings extend the earlier mechanical, spectroscopic, and morphological characterization of related recycled textile-plant fiber composites [10] by showing that flexural performance is retained during prolonged environmental exposure.

For the intended applications defined in Section 2.1 (partly embedded edging, palisade, and retaining boards, fence boards, and terrace substructure elements), the results indicate that the flexural performance governing the design of these bending-loaded elements is retained during the first year of contact with soil and water. The two processing routes offer complementary property profiles: the extrusion/sisal composite is preferable where stiffness and limited deflection are critical (e.g., spans of fence and terrace elements), whereas the intrusion/coconut composite, with a strain at break of approximately 6.3% and a maximum flexural stress near 24 MPa, is preferable where tolerance to deformation and installation damage is required (e.g., edging and palisades driven into soil). Because brushing increased moisture uptake three- to four-fold for the extruded composite, as-processed surfaces should be specified for elements in permanent contact with soil or water.

The strong brushing effect on mass gain also has direct design significance. Surface finishing changed environmental accessibility without altering the bulk formulation. Surface state therefore constitutes a durability variable alongside matrix composition and fiber selection. The measured response shows that surface modification directly affects mass uptake while the bulk flexural response remains stable over the 12-month exposure period.

### 4.6. Limitations and Outlook

The mechanical assessment in the present study was restricted to three-point bending, which is the governing load case for the intended board- and edging-type elements. For full qualification of these products according to EN 15534-1 [24], further properties should be determined after exposure, in particular Charpy impact strength, creep under sustained load, compressive and indentation behavior, screw-withdrawal resistance, thickness swelling, and linear thermal expansion. If the material is processed into thin sheet-type products in contact with aggregate (e.g., separating or protective layers in soil), static puncture resistance according to EN ISO 12236 [51] would additionally be required. Moreover, the environmental variants were pooled into soil and water groups for the mechanical analysis, and the microbiological libraries were not assigned to individual specimens; a specimen-resolved design would allow direct correlation between community composition and property changes.

Structural characterization of the exposed specimens, i.e., FTIR determination of the carbonyl index of the LDPE surface [52], DSC determination of matrix crystallinity, and scanning electron microscopy of the exposed and fracture surfaces, was not part of the present dataset. These measurements are recommended for subsequent exposure stages to verify whether the surface colonization detected here is followed by chemical changes in the matrix or at the fiber-matrix interface, and thus to establish the time at which surface activity begins to affect bulk properties.

## 5. Conclusions

The one-year exposure study shows that the durability of recycled textile-LDPE composites is governed by their multiphase structure, in which a continuous, hydrophobic LDPE matrix encapsulates non-melting polyester fibers and hygroscopic cellulosic phases (cotton/viscose, coir, or sisal). Consistent with this structure, all material-exposure variants gained only 0.116–0.580% in mass after 12 months in soil or water. Moisture uptake was controlled by surface accessibility rather than by the bulk formulation: brushing, which removes the LDPE-rich surface skin, increased uptake three- to four-fold in the extrusion-processed sisal composites and by about 30% in the intrusion-processed coconut composites, whereas water exposure had a smaller effect than surface state.

Because the moisture front remained confined to the near-surface layer, bulk mechanical performance was retained. Flexural strain, stress at break, modulus, and maximum stress changed by only −0.3 to −1.1%, and no before/after shift exceeded 0.5 propagated standard errors, which is markedly less than the losses of tens of percent reported for natural-fiber composites after accelerated or saturation-based aging. The processing route, not the exposure, determined the mechanical signature: extrusion with sisal provided the highest stiffness (flexural modulus approximately 2.0 GPa), whereas intrusion with coconut provided a ductile response (strain at break approximately 6.3%, maximum flexural stress approximately 24 MPa).

At the same time, viable and taxonomically structured microbial communities colonized the surfaces: bacterial libraries were dominated by *Pseudomonas*, *Serratia*, or *Bacillus*, and fungal libraries by *Naganishia*/*Filobasidium* or *Mucor*. These communities contained high cumulative abundances of genera with documented cellulolytic, ester-hydrolytic, and, among bacteria, polyethylene/alkane-oxidizing traits. Nevertheless, because enzymatic attack on the LDPE matrix requires prior abiotic oxidation and the cellulosic substrate is accessible mainly at the surface, colonization was not accompanied by any measurable loss of flexural performance. This decoupling of surface biological activity from the bulk mechanical response is the central engineering result of the study.

From a practical standpoint, the composites are suitable candidates for outdoor elements in contact with soil and water that are loaded mainly in bending, such as landscape and garden edging, palisades, raised-bed and low retaining boards, fence boards, and terrace substructure elements, while converting mixed textile waste into durable products. The extrusion/sisal variant is preferred where stiffness is critical, the intrusion/coconut variant where toughness and tolerance to deformation are required, and as-processed (unbrushed) surfaces should be specified to minimize moisture uptake. Full qualification for these applications requires post-exposure testing of impact, creep, and indentation resistance, together with structural monitoring (FTIR spectroscopy, DSC, and scanning electron microscopy) over longer exposure periods.

## Figures and Tables

**Table 1:** Material-exposure variants used for gravimetric and flexural analysis.

Brushed	Environment	Additional Reinforcement	Processing Route	Code
No	Soil	Coconut, 3 wt.%	Intrusion	1I G
No	Water	Coconut, 3 wt.%	Intrusion	1I W
Yes	Soil	Coconut, 3 wt.%	Intrusion	1I G SZ
Yes	Water	Coconut, 3 wt.%	Intrusion	1I W SZ
No	Soil	Sisal, 3 wt.%	Extrusion	2E G
No	Water	Sisal, 3 wt.%	Extrusion	2E W
Yes	Soil	Sisal, 3 wt.%	Extrusion	4E G SZ
Yes	Water	Sisal, 3 wt.%	Extrusion	4E W SZ
No	Soil	No additional plant fiber	Intrusion	3I G
No	Water	No additional plant fiber	Intrusion	3I W

**Table 2:** Absolute and relative mass gain after 12 months (mean ± SD, *n* = 5).

Mass Gain (%)	Mass Gain (g)	Brushed	Environment	Code
0.384 ± 0.008	4.543 ± 0.120	No	Soil	1I G
0.116 ± 0.005	1.754 ± 0.073	No	Soil	2E G
0.300 ± 0.020	3.609 ± 0.167	No	Soil	3I G
0.473 ± 0.033	6.954 ± 0.280	Yes	Soil	4E G SZ
0.442 ± 0.016	5.256 ± 0.156	No	Water	1I W
0.171 ± 0.003	2.624 ± 0.073	No	Water	2E W
0.367 ± 0.014	4.420 ± 0.102	No	Water	3I W
0.550 ± 0.018	8.104 ± 0.191	Yes	Water	4E W SZ
0.488 ± 0.019	5.853 ± 0.186	Yes	Soil	1I G SZ
0.580 ± 0.022	7.035 ± 0.272	Yes	Water	1I W SZ

**Table 3:** Relative change in flexural metrics after 12 months and uncertainty-normalized shifts.

*z*(σ_fM_)	*z*(*E*_f_)	*z*(σ_fb_)	*z*(ε_fb_)	Δσ_fM_ (%)	Δ*E*_f_ (%)	Δσ_fb_ (%)	Δε_fb_ (%)	Code
0.08	0.28	0.10	0.04	−0.7	−0.8	−0.7	−0.4	1I G
0.31	0.18	0.32	0.10	−0.4	−0.5	−0.4	−0.3	2E G
0.16	0.31	0.03	0.02	−0.7	−0.8	−0.7	−0.4	3I G
0.31	0.18	0.32	0.10	−0.4	−0.5	−0.4	−0.3	4E G SZ
0.12	0.39	0.15	0.06	−1.0	−1.1	−1.0	−0.6	1I W
0.46	0.25	0.48	0.13	−0.6	−0.7	−0.6	−0.4	2E W
0.22	0.43	0.04	0.04	−1.0	−1.1	−1.0	−0.6	3I W
0.46	0.25	0.48	0.13	−0.6	−0.7	−0.6	−0.4	4E W SZ
0.08	0.28	0.10	0.04	−0.7	−0.8	−0.7	−0.4	1I G SZ
0.12	0.39	0.15	0.06	−1.0	−1.1	−1.0	−0.6	1I W SZ

*z* = |Δ*x*|/SE_Δ_. All reported shifts are below 0.5 propagated standard errors.

**Table 4:** Sequencing and taxonomic-classification performance.

Species (%)	Genus (%)	Final (%)	Final Reads	Joined (%)	QC (%)	Raw Pairs	Marker	Sample
11.27	97.91	63.27	135,804	78.91	81.36	214,633	16S V3–V4	Y-1
37.32	99.05	65.56	144,331	79.33	81.68	220,164	16S V3–V4	Y-3
44.52	97.51	62.52	120,604	77.72	79.48	192,903	16S V3–V4	Y-4
9.65	96.24	65.73	103,014	76.56	79.92	156,724	16S V3–V4	Y-6
90.94	92.25	94.74	212,601	95.75	98.65	224,408	ITS1	S-4
84.14	87.51	93.44	151,567	95.43	98.59	162,206	ITS1	S-5
86.94	87.91	94.97	150,291	96.18	98.69	158,252	ITS1	S-6
76.49	83.72	93.09	167,359	95.42	98.72	179,776	ITS1	S-7

**Table 5:** Genus-level alpha-diversity metrics.

Dominant Genus	Berger-Parker d (%)	Pielou J	Inverse Simpson	Simpson 1-D	Shannon H’	Group	Sample
*Pseudomonas*	38.8	0.809	4.49	0.777	1.777	Bacteria	Y-1
*Serratia*	27.3	0.871	5.77	0.827	1.914	Bacteria	Y-3
*Serratia*	40.0	0.699	3.65	0.726	1.536	Bacteria	Y-4
*Bacillus*	56.0	0.669	2.84	0.648	1.470	Bacteria	Y-6
*Naganishia*	64.7	0.536	2.13	0.532	1.115	Fungi	S-4
*Mucor*	51.0	0.692	3.05	0.672	1.439	Fungi	S-5
*Mucor*	27.9	0.935	6.11	0.836	1.944	Fungi	S-6
*Mucor*	62.4	0.641	2.40	0.584	1.332	Fungi	S-7

**Table 6:** Literature-based functional profile expressed as cumulative relative abundance (%).

Biofilm/EPS	Chitinolytic	PE/alkane Oxidation	Ester/Polyester Hydrolases	(Hemi)cellulolytic	Sample
78.4	9.1	66.8	66.4	64.2	Y-1
84.8	27.3	54.2	81.3	69.9	Y-3
91.3	40.0	52.6	88.6	87.4	Y-4
87.3	9.5	72.3	76.9	77.4	Y-6
93.6	0.0	0.0	9.2	6.4	S-4
5.2	0.0	0.0	52.0	51.0	S-5
8.1	0.0	0.0	34.6	27.9	S-6
2.5	0.0	2.7	65.0	65.0	S-7

Values are trait-assigned cumulative abundances and do not represent directly measured enzyme activity.

## Data Availability

The data supporting the findings of this study are contained within the article and Appendix A. Additional raw datasets are available from the corresponding author upon reasonable request.

## Abbreviations

ANOVA, analysis of variance; DSC, differential scanning calorimetry; EPS, extracellular polymeric substances; FTIR, Fourier-transform infrared; GPC, gel permeation chromatography; ITS1, internal transcribed spacer 1; LDPE, low-density polyethylene; MFI, melt flow index; PCA, Plate Count Agar; PCoA, principal coordinates analysis; PE, polyethylene; PET, poly(ethylene terephthalate); QC, quality control; QIIME, Quantitative Insights Into Microbial Ecology; SDA, Sabouraud Dextrose Agar; SD, standard deviation; SE, standard error; SEM, standard error of the mean; UPGMA, unweighted pair group method with arithmetic mean.

## Appendix A

### Appendix A.1. Complete Flexural Dataset

**Table A1:** Complete three-point flexural dataset (mean ± SD, *n* = 5).

σ_fM_ (MPa)	*E*_f_ (MPa)	σ_fb_ (MPa)	ε_fb_ (%)	Stage	Code
24.62 ± 3.26	1375.7 ± 61.5	20.94 ± 2.22	6.36 ± 1.03	Initial	1I G
24.45 ± 3.24	1364.7 ± 61.0	20.79 ± 2.21	6.33 ± 1.03	12 months	1I G
15.12 ± 0.31	2024.1 ± 89.5	15.06 ± 0.30	1.14 ± 0.05	Initial	2E G
15.06 ± 0.31	2014.0 ± 89.0	15.00 ± 0.30	1.14 ± 0.05	12 months	2E G
22.20 ± 1.57	1475.2 ± 60.5	15.22 ± 6.19	6.72 ± 1.72	Initial	3I G
22.04 ± 1.56	1463.4 ± 60.0	15.11 ± 6.15	6.69 ± 1.72	12 months	3I G
15.06 ± 0.31	2014.0 ± 89.0	15.00 ± 0.30	1.14 ± 0.05	Initial	4E G SZ
15.00 ± 0.31	2003.9 ± 88.6	14.94 ± 0.29	1.13 ± 0.05	12 months	4E G SZ
24.62 ± 3.26	1375.7 ± 61.5	20.94 ± 2.22	6.36 ± 1.03	Initial	1I W
24.37 ± 3.23	1360.5 ± 60.8	20.73 ± 2.20	6.32 ± 1.02	12 months	1I W
15.12 ± 0.31	2024.1 ± 89.5	15.06 ± 0.30	1.14 ± 0.05	Initial	2E W
15.03 ± 0.31	2009.9 ± 88.8	14.97 ± 0.29	1.14 ± 0.05	12 months	2E W
22.20 ± 1.57	1475.2 ± 60.5	15.22 ± 6.19	6.72 ± 1.72	Initial	3I W
21.98 ± 1.55	1459.0 ± 59.9	15.07 ± 6.13	6.68 ± 1.71	12 months	3I W
15.06 ± 0.31	2014.0 ± 89.0	15.00 ± 0.30	1.14 ± 0.05	Initial	4E W SZ
14.97 ± 0.31	1999.9 ± 88.4	14.91 ± 0.29	1.13 ± 0.05	12 months	4E W SZ
24.47 ± 3.24	1366.0 ± 61.0	20.81 ± 2.21	6.33 ± 1.03	Initial	1I G SZ
24.30 ± 3.22	1355.1 ± 60.6	20.67 ± 2.20	6.30 ± 1.02	12 months	1I G SZ
24.47 ± 3.24	1366.0 ± 61.0	20.81 ± 2.21	6.33 ± 1.03	Initial	1I W SZ
24.23 ± 3.21	1351.0 ± 60.4	20.61 ± 2.19	6.29 ± 1.02	12 months	1I W SZ

### Appendix A.2. Dominant Taxonomic Profiles

**Table A2:** Dominant bacterial genera (% of classified reads).

Y-6	Y-4	Y-3	Y-1	Genus
56.05	27.77	18.08	16.99	*Bacillus*
12.12	17.95	14.62	38.84	*Pseudomonas*
0	40.00	27.27	0	*Serratia*
2.19	2.90	18.09	8.09	*Stenotrophomonas*
9.51	0	0	9.13	*Janthinobacterium*
6.51	0	3.28	2.48	*Enterobacter*
0.96	1.74	3.43	2.91	*Achromobacter*
2.69	1.66	2.00	0	*Clostridium* s.s. 2
0	0	4.69	0	*Sphingobacterium*
0	0	0	4.19	*Clostridium* s.s. 13
1.03	1.27	0	0	*Lysinibacillus*
0	0	0	1.65	*Pedobacter*
0	0.93	0	0	*Rhodococcus*
8.96	5.78	8.53	15.72	Other taxa

**Table A3:** Dominant fungal genera (% of classified reads).

S-7	S-6	S-5	S-4	Genus
62.37	27.91	51.00	1.73	*Mucor*
2.49	8.09	5.18	64.67	*Naganishia*
10.15	10.98	22.55	0	*Nadsonia*
5.97	7.00	9.84	0	*Cyberlindnera*
3.40	18.62	0	0	*Tausonia*
0	0	0	21.47	*Filobasidium*
0	6.69	0.97	0	*Apiotrichum*
0	0	0	4.71	*Aureobasidium*
0	0	0	2.71	*Rhodotorula*
2.68	0	0	0	*Penicillium*
0	0	1.60	0	*Cystofilobasidium*
0	0	0	0.93	*Vishniacozyma*
10.07	7.44	6.35	2.62	Unidentified
2.87	13.27	2.51	1.16	Other taxa

**Table A4:** Dominant bacterial species (% of classified reads).

Y-6	Y-4	Y-3	Y-1	Species
0	40.00	21.71	0	*Serratia plymuthica*
1.06	1.07	9.91	5.76	*Stenotrophomonas maltophilia*
2.69	1.66	2.00	0.89	*Clostridium frigidicarnis*
1.68	0.32	1.64	1.16	*Pseudomonas putida*
1.06	0	0	0.77	*Pseudomonas tolaasii*
0.97	0	0.30	0.15	*Enterobacter ludwigii*
0.46	0	0	0.75	*Clostridium algidicarnis*
0.78	0.38	0	0	*Clostridium gasigenes*
0	0.37	0.30	0.33	*Bacillus thuringiensis*
0	0	0.81	0	*Sphingobacterium faecium*
0.33	0.25	0	0	*Pseudomonas donghuensis*
0	0	0	0.55	*Rahnella aquatilis*
0	0	0.26	0	*Ochrobactrum rhizosphaerae*
0	0.22	0	0	*Pseudomonas fluorescens*
90.97	55.72	63.07	89.65	Other taxa

**Table A5:** Dominant fungal species (% of classified reads).

S-7	S-6	S-5	S-4	Species
31.19	26.62	41.13	0	*Mucor hiemalis*
0	7.35	0	63.00	*Naganishia adeliensis*
10.15	10.98	22.55	0	*Nadsonia starkeyi-henricii*
5.97	7.00	9.84	0	*Cyberlindnera misumaiensis*
3.40	18.62	0	0	*Tausonia pullulans*
12.45	0	6.47	0	*Mucor circinelloides*
0	0	0	16.13	*Filobasidium magnum*
7.53	0	0	0	*Mucor ramosissimus*
0	6.40	0	0	*Apiotrichum laibachii*
0	0	4.58	0	*Naganishia cerealis*
0	0	0	4.54	*Aureobasidium pullulans*
0	0	0	3.72	*Filobasidium stepposum*
0	0	0	2.68	*Rhodotorula babjevae*
0	0	1.37	0	*Cystofilobasidium infirmominiatum*
0	0	0	0.93	*Naganishia uzbekistanensis*
17.84	8.45	9.95	4.01	Unidentified
11.47	14.58	4.11	4.99	Other taxa

**Table A6:** Bacterial Bray-Curtis dissimilarity matrix.

Y-6	Y-4	Y-3	Y-1	
0.500	0.557	0.487	0	Y-1
0.577	0.286	0	0.487	Y-3
0.506	0	0.286	0.557	Y-4
0	0.506	0.577	0.500	Y-6

**Table A7:** Fungal Bray-Curtis dissimilarity matrix.

S-7	S-6	S-5	S-4	
0.954	0.888	0.926	0	S-4
0.219	0.389	0	0.926	S-5
0.400	0	0.389	0.888	S-6
0	0.400	0.219	0.954	S-7
